# HE4 as a Biomarker for Endometrial Cancer

**DOI:** 10.3390/cancers13194764

**Published:** 2021-09-23

**Authors:** Roya Behrouzi, Chloe E. Barr, Emma J. Crosbie

**Affiliations:** 1 Department of Medical Oncology, The Christie NHS Foundation Trust, Manchester M20 4BX, UK; rbehrouzi@doctors.org.uk; 2 Manchester Academic Health Science Centre, Department of Obstetrics and Gynaecology, St Mary’s Hospital, Manchester University NHS Foundation Trust, Manchester M13 9WL, UK; Chloe.Barr@mft.nhs.uk; 3 Gynaecological Oncology Research Group, Division of Cancer Sciences, University of Manchester, Manchester M13 9WL, UK

**Keywords:** human epididymis protein 4, HE4, endometrial cancer, atypical endometrial hyperplasia, biomarker

## Abstract

**Simple Summary:**

There are currently no blood biomarkers approved for routine clinical use in endometrial cancer. Serum human epididymis protein 4 (HE4) is significantly higher in patients with endometrial cancer compared to patients without endometrial cancer and is associated with a poorer prognosis. This makes HE4 an attractive candidate for clinical use in endometrial cancer. The aim of this review is to summarise the evidence for the use of serum HE4 in the detection, prognosis, prediction of therapy response and recurrence monitoring in endometrial cancer. The utility of combining HE4 with other biomarkers or imaging and clinical variables, and its detection in other biofluids is also discussed, as well as potential challenges for clinical use and recommended areas for future research.

**Abstract:**

There are currently no blood biomarkers in routine clinical use in endometrial carcinoma (EC). Human epididymis protein 4 (HE4) is a glycoprotein that is overexpressed in the serum of patients with EC, making it a good candidate for use as a diagnostic and/or prognostic biomarker. HE4 is correlated with poor prognostic factors, including stage, myometrial invasion and lymph node metastases, which means it could be used to guide decisions regarding the extent of surgery and need for adjuvant therapy. Serum HE4 has also shown promise for predicting responses to progestin therapy in early-stage EC. The use of algorithms and indices incorporating serum HE4 and other biomarkers, including clinical and imaging variables, is an area of increasing interest. Serum HE4 levels rise with age and renal dysfunction, which may affect the interpretation of results. This review covers the evidence supporting the use of HE4 as an EC biomarker for diagnosis, prognosis, recurrence monitoring, and prediction of therapy response. The evidence for combining serum HE4 with other biomarkers, including clinical and imaging variables, its value as a biomarker in other biofluids and potential challenges of its clinical use are also discussed.

## 1. Introduction

Endometrial carcinoma (EC) is the most common gynaecological malignancy. Its incidence has risen by 55% over the last 30 years, with the death rate also increasing by 23% [1]. This is attributable to the rising incidence of obesity, which is estimated to contribute to up to 50% of cases [2], an ageing population, and a trend towards the medical management of benign gynaecological conditions with fewer hysterectomies [3,4]. Around 90% of women with EC present with abnormal uterine bleeding, and an increasing number are pre-menopausal [5]. Investigations include transvaginal ultrasound (TVUS), endometrial biopsy and in some cases outpatient hysteroscopy. These investigations have a good sensitivity for the diagnosis of EC but are limited by poor specificity, as is the case with TVUS, or are invasive and painful [6]. Overall, EC has an excellent five-year survival of 84%, since two-thirds of cases present at an early, curable stage [1]. However, the prognosis for women who present with high risk or advanced disease remains extremely poor.

The mainstay of treatment for EC is a total hysterectomy and bilateral salpingo-oophorectomy, and many women also require adjuvant therapy to reduce the risk of recurrence. In some women, surgical management is not safe or is inappropriate. This includes women with class III obesity (body mass index > 40 kg/m^2^) and/or medical co-morbidities with high risk of surgical morbidity and mortality, and those wishing to preserve fertility. In these cases, women are managed with primary radiotherapy or hormone therapy with progestin. The decision regarding primary treatment and the recommendations for adjuvant therapy are based on the traditional Bokhman dualistic model and prognostic histopathological features of the tumour [7]. However, around 13–17% of women with EC will still recur despite risk stratification [8].

There are currently no diagnostic or prognostic blood biomarkers in routine clinical use for EC. There are also no blood biomarkers approved for predicting response to systemic treatment or for monitoring for recurrence in EC post radical treatment. The optimum biomarker for EC would have a high sensitivity and specificity for detecting EC compared to benign and healthy controls. The ideal receiver-operating characteristic area under curve (AUC) would be close to 1, with minimum of 0.7 to indicate clinical utility as a biomarker [9]. A blood biomarker is relatively non-invasive compared to tissue biomarkers, which require either a biopsy or surgical specimen, and could also be used at multiple points in diagnostic and treatment pathways with less associated pain and anxiety. An accurate diagnostic biomarker deployed in primary care could reduce the number of women referred for painful and costly investigations. A prognostic biomarker could help risk stratify women with EC to aid surgical planning, decisions about adjuvant treatment, follow up programmes and monitoring for recurrence, creating a more personalised approach to management. A predictive biomarker could also help guide decisions regarding systemic therapy, such as conservative management with progestin in early-stage EC. Indeed, these important clinical uses have not only been identified by clinicians, but also by EC patients and carers as areas worthy of further development in our recent James Lind Alliance research gap analysis [10,11].

Human epididymis protein 4 (HE4) is a whey acidic protein that was first identified in the epithelium of the distal epididymis [12]. It is encoded by the WFDC2 gene on chromosome 20q12-13.1 and contains a WAP-type four disulphide core domain with a sequence homologous to extracellular proteinase inhibitors. It is expressed in the epithelium of several tissues, including the female reproductive tract, and is overexpressed in a variety of cancers [13]. The biological function of HE4 is unclear, although recent studies have shown that HE4 enhances EC proliferation, invasion, and growth [14,15]. Serum HE4 is currently licensed for use in the diagnosis and monitoring of recurrence in ovarian cancer. There is increasing interest in HE4 as a biomarker for EC, since HE4 is overexpressed in >90% of ECs [13]. HE4 has demonstrated superior sensitivity and specificity compared to serum cancer antigen 125 (CA125) for detecting EC, and has been found to correlate with histopathological markers of disease severity, survival and recurrence, making it a promising non-invasive biomarker [16].

The aim of this review is to summarise the evidence supporting the role of HE4 as a diagnostic, prognostic and predictive biomarker for EC, both alone and in combination with other biomarkers, and its potential utility in clinical practice. To identify relevant studies, the Medline, Embase and Cochrane library were systematically searched from database inception to July 2021 for English language articles using the following keywords: “endometrial cancer”, “endometrial carcinoma”, “endometrial hyperplasia”, “atypical endometrial hyperplasia” associated with “HE4”, “human epididymis protein 4”, “WFDC2”, “WAP Four-Disulfide Core Domain 2”. Only original clinical research articles and meta-analyses were used for data extraction.

## 2. HE4 as a Diagnostic Biomarker

Currently, in the UK, all women presenting to their General Practitioner (GP) with suspected EC are referred to secondary care for further investigations. Presentation to the gynaecology clinic with post-menopausal bleeding is extremely common, but only around 5% have an underlying endometrial malignancy, leading to unnecessary discomfort, pain and anxiety in the majority of those investigated, and additional costs to the healthcare service [6]. Women undergo a TVUS to assess endometrial thickness, the sensitivity of which is 98%, 95% and 90% at cut-offs of 3 mm, 4 mm and 5 mm, respectively. Whilst the sensitivity of TVUS is excellent, its use is limited by poor specificity, as benign pathologies such as polyps and fibroids may create the appearance of a thickened endometrium [17]. Those with an endometrial thickness above the threshold are recommended to undergo outpatient endometrial sampling. Endometrial biopsy has an excellent sensitivity of 99% [18], but many women find the experience unacceptably invasive and painful. There are also high rates of failed sampling due to cervical stenosis, and around a third have a biopsy taken that is inadequate for diagnosis [19]. Outpatient hysteroscopy is carried out when outpatient endometrial biopsy is not possible or if there are irregularities on ultrasound indicating a high risk for EC [17]. In addition to discomfort and pain, hysteroscopy poses a risk of bleeding, infection, and uterine perforation, which can be life threatening. Where outpatient hysteroscopy has failed or is poorly tolerated, women are required to undergo the procedure under a general anaesthetic, further increasing their morbidity risk and time to diagnosis. Whilst outpatient procedures are accurate diagnostic tools, they are associated with unacceptable discomfort and anxiety, with up to 34% reporting severe pain [20,21].

Several studies have investigated the performance of serum HE4 as a diagnostic biomarker for EC and have shown that serum HE4 is elevated in women with EC compared to healthy and benign gynaecological controls. In a meta-analysis by Li et al. of 23 studies, serum HE4 had a pooled sensitivity, specificity and AUC of 65%, 91% and 0.84, respectively, in diagnosis of EC compared to healthy or benign controls [22]. Liu et al. had similar results from 17 studies with a pooled sensitivity, specificity and AUC of 65%, 91% and 0.75, respectively [23]. This suggests serum HE4 has a high specificity and moderate accuracy for EC diagnosis, and therefore good potential as a diagnostic biomarker, although its clinical utility may be limited by the lower sensitivity. A further meta-analysis by Li et al. demonstrated similar findings from 12 studies, reporting a pooled sensitivity of 71%, a specificity of 87% and an AUC of 0.88. HE4 also had superior diagnostic ability compared to CA125 in EC, which had a reported AUC of only 0.58, indicating serum HE4 has a good potential for use in diagnosis of EC compared to CA125 [24]. Whilst the three meta-analyses support the potential use of HE4 as a diagnostic marker, all report significant heterogeneity, which limits the findings.

Studies have also shown that serum HE4 levels do not differ significantly in healthy controls compared to those with benign gynaecological conditions, including benign tumours and endometriosis [25,26]. In the post-menopausal population, Dewan et al. reported a sensitivity and specificity of serum HE4 to diagnose EC from healthy controls of 87% and 100% respectively [27], supporting the diagnostic potential of HE4 in the predominantly target population.

Atypical endometrial hyperplasia (AEH) is a precursor lesion of endometrioid EC, and its presentation and risk factors are the same. Due to the high risk of progression to EC, the recommended management is a total hysterectomy. An estimated 40% of women diagnosed with AEH on endometrial biopsy also have a concurrent early-stage EC [28]. A small number of studies have investigated serum HE4 levels in women with AEH and have shown increased serum concentrations in AEH compared to endometrial hyperplasia without atypia (EH) [29,30]. Yilmaz et al. demonstrated a higher concentration of serum HE4 in women with AEH compared to both EH (71 pM vs. 36 pM, *p* < 0.01) and healthy controls (71 pM vs. 46 pM, *p* = 0.005) [31].

Overall, the evidence supports serum HE4′s potential as an effective biomarker for differentiating malignant endometrium from both benign and normal endometrium. Furthermore, studies have shown that serum HE4 is higher in patients with early-stage EC (stage I/IA) compared to those with benign endometrial pathologies [31,32], or healthy controls [26], making it a promising biomarker for early detection, and one that could be used in primary care to triage referrals. The high specificity reported in the literature could make this biomarker useful in combination with TVUS, improving the overall specificity of endometrial thickness, reducing the number of women requiring endometrial biopsy and outpatient hysteroscopy.

Additionally, serum HE4 may have a potential role in screening. Whilst there is no evidence to support screening for EC in the general population using TVUS and endometrial sampling, annual screening or prophylactic hysterectomy is recommended for women at high risk of EC, such as those with Lynch syndrome [17]. Lynch syndrome is an inherited autosomal dominant condition caused by a mutation in a DNA mismatch repair gene, predisposing individuals to a number of cancers including colon, endometrial and ovarian cancers, with up to 60% lifetime risk of EC [33]. For some women with Lynch syndrome, a prophylactic hysterectomy is not an acceptable option due to wishes to preserve fertility, and screening using TVUS and endometrial biopsy is invasive and painful. Screening with serum HE4 may provide an attractive alternative.

Despite promising results, the sensitivity of serum HE4 varies widely between studies. This may be due to several reasons. First, all the studies have small sample sizes. Second, there is heterogeneity in the cut-off value used to evaluate the diagnostic ability of HE4 for EC. Third, the studies use different control groups, making comparison challenging. Future multicentre studies are awaited which will look at serum HE4 as a diagnostic biomarker prospectively [34]. Table 1 summarises the cut-off values for HE4 used in different studies for the diagnosis of EC and their performance.

## 3. HE4 as a Prognostic Marker

The majority of women with EC present at an early stage of disease with an excellent five-year survival of 92% [1]. However, around a fifth present with advanced stage disease which has a poorer prognosis and five-year survival of 15–48% [1]. The management of early-stage EC is surgical and includes a total hysterectomy and bilateral salpingo-oophorectomy. Some women may require more extensive surgery including lymphadenectomy and omentectomy depending on tumour stage, grade and histological subtype. Pre-operative surgical planning is guided by endometrial histology and provisional staging by CT scan to rule out metastatic disease in patients with high risk histologies. An MRI scan is superior to CT for the assessment of myometrial invasion, an important determinant of suitability for non-surgical management in women wishing to preserve their fertility. However, due to limitations of clinical staging, final histological diagnosis and staging is based on the surgical specimen. An accurate blood biomarker that could aid the detection of deep myometrial invasion (MI) and lymph node metastases (LNM) pre-operatively would improve surgical planning with a potential impact on survival. The decision to offer adjuvant treatment with radiotherapy +/− chemotherapy is based on the traditional dualistic model and pathological prognostic features [7,17]. However, around 20% of women with type I EC recur whereas around 50% of those with type II EC do not [50], suggesting some women who may benefit from adjuvant therapy are not receiving it, whereas others may be receiving potentially harmful adjuvant therapy unnecessarily.

There are several clinicopathological factors that are known to be independently associated with EC prognosis, including stage at diagnosis, histological subtype, grade, depth of MI and the presence of lympho-vascular space invasion (LVSI). Traditionally, EC has been divided into two groups based on the Bokhman classification [51]. Type I EC includes low grade, early-stage, endometrioid tumours, with no LVSI and <50% MI. They tend to be hormone driven with an excellent prognosis. In contrast, type II EC includes high grade subtypes, such as serous and clear cell tumours, which are more often advanced stage at diagnosis, with high rates of LVSI and MI >50%. They have a more aggressive course and are not hormonally driven. Over the last decade, it has become clear that EC is a more heterogenous disease than previously thought. In 2013, the TCGA described a new classification of EC based on the tumour’s molecular and genomic profile, into four distinct groups: POLE ultra-mutated (POLEmut), microsatellite instability hypermutated, copy-number low and copy-number high [52]. Each group has a different prognosis, highlighting differing prognostic profiles amongst tumours with the same histological subtype and grade, supporting the theory that some women may currently be over-treated, and others undertreated. An international consortium has developed a more clinically applicable and cost-effective model based on the TCGA classification. The TransPORTEC classification groups endometrial tumours into POLEmut, mismatch repair deficient (MMRd), p53 abnormal (p53abn) and no specific molecular profile (NSMP), and each of these groups has the same prognostic profile described by the TCGA groups [53]. There has been increasing research investigating the role of molecular classification for risk stratification of women with EC to aid decisions on management and follow up programmes, and recent updates in international EC guidance now reflect this [7]. It is likely that over the coming years, molecular classification will become the ‘gold standard’ prognostic marker for EC. Evidence suggests that the POLEmut group have an excellent survival receiving minimal benefit from adjuvant therapy, whereas the p53abn group have a poor prognosis, and derive significant benefit. A limitation to this classification is that for the MMRd and NSMP groups, who have almost overlapping overall survival (OS) curves and a moderate prognosis, the benefit of adjuvant therapy is unclear [54]. A blood biomarker may help further risk stratify women within these two groups to individualise treatment. Furthermore, molecular classification requires a tissue biopsy or a surgical specimen, and therefore may not be appropriate for pre-operative surgical planning.

Pre-surgically, MRI is the imaging modality of choice for EC assessment. However, its ability to detect deep MI, LNM and cervical involvement (CI) varies widely, with studies reporting sensitivities of 60–88%, 71% and 41% respectively [55,56,57]. Microscopic nodal metastasis and superficial cervical mucosal involvement are both difficult to identify pre-operatively, and large or polypoidal tumours, endometrial cavity distension and fibroids all contribute to poor detection of deep MI [57]. Studies have shown that 22–33% of women with stage IA disease were upstaged at final histology, with deep MI diagnosed in 33% and pelvic nodal involvement in 8.2% of those with grade 1 disease [55,58].

There is growing evidence that HE4 may be useful as a prognostic marker in EC, with many studies showing an association between serum HE4 and poor prognostic histopathological factors, including ≥50% MI [27,31,35,42,55,59,60,61,62,63,64,65,66,67,68,69,70], CI and stage [42,60,65,66,69], presence of LVSI [31,42,55,60,64,66,68,69], tumour size [31,55,67,68,69,70] and LNM [27,31,32,44,55,60,64,65,68,69]. Capriglione et al. demonstrated that the proportion of EC patients with a cut-off of HE4 >70 pM was 42%, 77%, 90%, 93% and 100% at stages IA, IB, II, III and IV, respectively, and suggested ideal serum HE4 cut-offs by stage with >80% sensitivity and >95% specificity [71]. Serum HE4 is also associated with increased endometrial thickness [31] and tumour free distance to serosa ≤ 7 mm [66]. More advanced features including ascites [44], peritoneal positive cytology [60] and extrauterine metastases [55] are similarly associated with higher serum HE4 levels.

Whilst endometrial biopsy is excellent at diagnosing EC, the concordance between pre-operative and post-operative grading is poor. This has been shown to differ by grade, with grade 1 having 73% concordance but grades 2 and 3 only having 52% and 53%, respectively [72]. The evidence regarding the association of HE4 and tumour grade is more controversial, with some studies reporting serum HE4 increases with tumour grade [26,29,31,32,39,59,60,65,67,71,73], and others showing no significant difference [47,61,62,74]. Patients with low risk endometrioid EC with primary tumour diameter ≤2 cm and MI ≤50% had a significantly lower serum HE4 compared to all other type I ECs [61].

The incidence of LNM increases with increasing grade, but there is currently little evidence that routine lymphadenectomy in those with early-stage, low grade EC improves outcomes [17,75,76]. However, there is growing support for sentinel lymph node mapping to predict LNM for the purpose of surgical staging [77,78]. Considering the poor concordance between pre- and post-operative histology and the limitations of MRI, some patients may be under-staged, affecting their chances of receiving adjuvant therapy. HE4 is associated with LNM and may aid in pre-operative surgical planning. Dobrzycka et al. ratified patients with early-stage endometrioid EC who required lymphadenectomy (stage IA, G3, IB and II) and those who did not (stage IA, G1 and G2) and found those who required lymphadenectomy had a higher serum HE4 pre-operatively [73]. Gasiorowska et al. also showed that patients with EC who needed lymphadenectomy had significantly higher serum HE4 than those with no indications for lymphadenectomy (i.e., stage IA, G1–2) [39].

There have been mixed results from studies regarding the relationship between serum HE4 and histological subtypes of EC. A number of studies have shown no difference in levels of serum HE4 [27,32,61,62,63,64,74] between patients with different subtypes including endometrioid vs. non-endometrioid histology. Some studies have shown that serum HE4 is higher in endometrioid EC compared to non-endometrioid EC [35]. Kalogera et al. found that there was no difference in serum HE4 levels in patients with type I compared to type II ECs [61]. In contrast, other studies have shown patients with non-endometrioid EC have a higher serum HE4 than endometrioid EC [31,71]. For example, two studies showed that patients with serous histology had significantly higher serum HE4 compared to those with endometrioid histology [30,39]. The conflicting reports of serum HE4 levels in endometrioid vs. non-endometrioid EC raise the possibility that serum HE4 may not be a reliable marker for differentiating between different pathological subtypes of EC, although the variation of results may be due to the greater prevalence of endometrioid EC compared to non-endometrioid EC and limited sample sizes in studies.

Serum HE4 is strongly associated with survival in patients with EC. A meta-analysis by Dai et al. looked at 29 studies with 4235 patients and reported higher levels of HE4 were significantly associated with worse OS (HR 2.15, 95%CI 1.11–2.62, *p* < 0.001), disease free survival (DFS) (HR 2.50, 95%CI 1.86–3.37, *p* < 0.001) and progression free survival (PFS) (HR 1.27, 95%CI 1.11–1.45, *p* = 0.001) [79]. Bignotti et al. showed that higher serum HE4 before treatment was associated with shorter OS (*p* = 0.020) and shorter PFS (*p* = 0.03) in patients with EC [60]. Mutz-Dehbalai et al. showed that raised HE4 was associated with reduced OS over a median follow up of three years. Patients with a baseline HE4 ≥ 81 pM had a lower five-year OS rate of 60% compared to 86% (*p<* 0.001) in those with HE4 < 81 pM [62]. Serum HE4 ≥ 81 pM was also independently prognostic of OS when adjusted for age, stage, histology and grade (HR 2.40, 95%CI 1.17–4.97, *p* = 0.017). Other studies have similarly shown serum HE4 is an independent predictive biomarker of OS when adjusted for age, FIGO stage, grade, MI, LNM and LVSI [64,66]. Another study of patients with EC receiving a median follow up of 48 months post-surgery showed that the median HE4 in the nine (6%) patients that died was significantly higher at 109 pM compared to 77 pM in survivors [68]. Insin et al. showed that three-year OS was poorer (71% vs. 96%) in patients with pre-operative serum HE4 ≥ 70 pM who had surgery [80]. Cymbaluk-Ploska et al. also showed that baseline serum HE4 < 70 pM correlated with better OS and DFS [81].

Whilst much of the literature suggests a strong relationship between serum HE4 and high-risk features, many of the studies are limited by small numbers and heterogeneity in study design and inclusion criteria, such as differences in EC stage and histological subtypes, the serum threshold of HE4 and retrospective design. Further adequately powered prospective clinical studies are required to demonstrate the true benefit of HE4 as a prognostic marker. Nonetheless, it remains that HE4 has the potential to help predict women who are at high risk of progression and relapse, and help guide radical treatment decisions including lymphadenectomy, adjuvant radiotherapy and chemotherapy, and may be of particular use in combination with molecular markers. Table 2 summarises the serum cut-offs for HE4 used by different studies for various prognostic factors and their performance.

## 4. HE4 as a Biomarker for Therapy Response

For some women, standard management of EC by surgical intervention and adjuvant therapy may not be an option. The incidence of EC among young, pre-menopausal women is increasing, particularly for those with polycystic ovary syndrome, subfertility and obesity. In this population, a hysterectomy for the management of either AEH or an early-stage low grade EC may be undesirable, as many wish to retain their uterus for fertility preservation. Surgical management may also pose unacceptable peri-operative risks in women with class III obesity and associated medical co-morbidities and presents a number of challenges [87]. This group commonly suffer multiple related health conditions, including diabetes, hypertension and heart disease, requiring intensive pre-operative assessment, and often result in higher peri- and post- operative complications, longer hospital stay and higher treatment costs [88]. High dose oral or intrauterine progestin may be used as an alternative management option in women wishing to retain fertility or those unfit to undergo hysterectomy. It is recommended that only women with low-risk features of EC, such as grade 1, endometrioid subtype and <50% MI, in whom hysterectomy is contraindicated or undesired, be considered for conservative management [7,89,90]. There are no large randomised trials of progestin therapy investigating the optimal route, duration and dose of treatment, but there are a number of prospective studies evaluating progestin therapy in patients with early-stage EC or hyperplasia [91,92,93,94]. Evidence suggests favourable oncological and reproductive outcomes, with reported response rates of 45–70% in patients with Stage 1a EC and 65–90% in patients with AEH [91,92,93,94,95,96]. Despite this, there is a proportion of women who do not respond to progestin therapy, and there are high rates of recurrence once therapy is discontinued, leading to many inevitably having to undergo hysterectomy. Some studies have attempted to identify clinicopathological markers that predict response such as uterine size, baseline BMI, weight loss during treatment and age, but the evidence is conflicting [94,97,98,99,100]. A reliable non-invasive biomarker that could identify women who are more likely to respond to progestin therapy would improve the planning and counselling of women for conservative management, provide a more individualised treatment plan, and reduce the need for invasive endometrial biopsies and the risk of progression in those who are unlikely to respond [101,102].

Few studies have investigated HE4 as a predictive biomarker for therapy response. However, the promising association of HE4 with prognostic features of EC suggests that it may have a role. Orbo et al. showed a greater proportion of patients with hyperplasia (with or without atypia) who responded to progesterone therapy (oral or LNG-IUS) displayed a reduction in endometrial tissue HE4 expression at six months compared to those that did not respond (53% vs. 6%). In addition, a greater proportion of non-responders compared to responders had same/increased tissue HE4 expression after six months (95% vs. 49%) [103]. Only one study, by Behrouzi et al., has investigated serum HE4 as a predictive biomarker for progestin therapy response in AEH and endometrioid EC [96]. In this study we showed that higher baseline serum HE4 was predictive of poor response to intrauterine progestins over 12 months in 49 patients with AEH or stage IA endometrioid EC, and this remained significant after adjustment for age, grade, BMI, menopausal status, and histological subtype [96]. A greater proportion of responders compared to non-responders to the levonorgestrel-releasing intrauterine system (LNG-IUS) (71% vs. 36%) had a baseline serum HE4 < 70 pM [96]. A serum HE4 cut-off of ≥165 pM had a 100% specificity and 39% sensitivity for predicting resistance to the LNG_IUS, with AUC 0.76 (Table 3). There was a significant reduction in serum HE4 observed between baseline and three months in responders, which remained the case when considering % change in BMI as a covariable, although changes in serum HE4 from baseline over the treatment period were not overall predictive of response. Serum HE4 may therefore be an effective non-invasive marker that predicts response to progestin therapy. However, the above finding would require further validation in a larger cohort of patients to determine the most appropriate serum HE4 cut-off values for stratifying patients.

## 5. HE4 as a Biomarker for Recurrence

Around 13–17% of patients with EC suffer recurrence [104]. EC recurrence is more challenging to treat and has a poor OS compared to primary diagnosis, and survival is dependent on site of recurrence. Vaginal recurrence has an estimated three-year OS of around 73%, whereas three-year survival with distant recurrence is only around 15% [105]. Follow up programmes have traditionally been clinician-led, with all women attending the hospital for assessment at three-monthly intervals for the first three years, followed by six-monthly intervals for one year and then a final annual visit. The aims of follow up include the earlier detection of recurrence so effective treatment might be commenced, to identify and treat adverse effects of primary treatment, and to offer psychosocial support. Visits should include symptom enquiry, clinical examination and patient education regarding symptoms [17]. However, the optimal follow up programme for EC is unknown, and current evidence regarding the benefits is controversial, and there is evidence that up to 60% of recurrence may occur in women with low risk disease [106]. Around 70–80% of women present with symptomatic recurrence, namely vaginal bleeding, abdominal pain and discharge, and have a poorer OS compared to those with asymptomatic recurrence [106,107]. However, both lead and length time bias may induce an artificial survival advantage, and most studies are retrospective. Many gynaecological oncology centres are now offering women with low-risk EC patient initiated follow up (PIFU), whereby patients are informed of red flag symptoms and advised to contact secondary care, where they have open access. However, PIFU relies on the woman’s ability to detect recurrence, which is influenced by education level and socioeconomic status [107,108], and may increase fear of recurrence in EC survivors [107]. Currently, there is a lack of evidence to support routine diagnostic interventions such as biomarkers and imaging to detect recurrence at follow up [17]. Over the coming years there is likely to be a role for molecular classification in risk stratifying women for recurrence monitoring, therefore allowing for a more personalised approach to follow up, and an accurate serum biomarker may improve risk stratification and facilitate monitoring for recurrence.

As previously discussed, higher levels of serum HE4 are associated with known EC prognostic factors and worse OS, and so it is reasonable to hypothesise that it might have a role in predicting and monitoring for recurrence. A number of studies have demonstrated a higher serum HE4 at primary treatment is associated with shorter recurrence free survival (RFS) [60,62,64,104,109]. Brennan et al. showed that higher baseline serum HE4 was independently associated with reduced RFS after adjusting for stage and grade (HR 2.40, 95% CI 1.19–4.38, *p* = 0.014) over a median follow up of 37 months [104]. Similarly, Stiekema et al. found that serum HE4 was a strong independent prognostic factor for RFS in a cohort of 88 women, with a HR of 5.12 per 10-fold increase in HE4 (95%CI 1.54–17.1, *p* = 0.008) following adjustment for age, FIGO stage, grade, MI, LNM and LVSI [66]. The strong association with RFS supports the potential use of serum HE4 in risk stratification models and in individualising follow up.

Serum HE4 may also have a role in monitoring for recurrence. It has been shown that serum HE4 levels decrease significantly seven days after surgery compared to baseline pre-surgery [29], indicating a response to tumour removal and suggesting that increasing levels may be associated with tumour recurrence. Angioli et al. monitored serum HE4 every three months for two years, and then every six months up to five years, in patients who had radical treatment for EC and found serum HE4 at diagnosis of recurrence or the last recorded follow up was significantly higher in patients with recurrence (212 pM vs. 76 pM *p* = 0.03) [109]. In a study by Brennan et al., EC recurrence was detected by serum HE4 with an AUC of 0.81 (95%CI 0.71–0.90), and for endometrioid tumours, HE4 was superior to CA125 (AUC 0.81 (95%CI 0.79–0.95) vs. AUC 0.67 (95%CI 0.52–0.83) *p* = 0.017) [104]. Using a cut-off of 70 pM, Abbink et al. found that serum HE4 could detect recurrence at a median of 126 days before clinical confirmation of recurrent disease [64], supporting the idea that serum HE4 might have a promising role in monitoring for recurrence post treatment and might allow earlier detection of relapse. Bednarikova et al. showed that serum HE4 levels were higher at diagnosis and time of recurrence in patients with recurrence vs. those with remission, with a median follow up of 29.5 months after surgical resection for predominantly early-stage EC. However, adjuvant treatment was variable, with 35% receiving radiotherapy and 11% chemotherapy, and only a small proportion (five in 65) recurred [110]. Table 4 summarises the serum cut-offs for HE4 used by Brennan et al. and Angioli et al. for EC recurrence and their performance [104,109].

## 6. HE4 in Other Biofluids

Over the last few years, alternative sources of biomarkers are gaining interest, in particular urine. Urine has several benefits over blood as it lends itself to self-collection, is non-invasive, cheap and readily available, and urinary protein concentrations are more stable than serum concentrations [111]. Urinary biomarkers can include metabolites, proteins and peptides, extracellular microRNAs and tumour cells, several of which have been investigated for EC diagnosis [112]. Protein biomarkers are thought to enter the urine by excretion through the kidney or urinary contamination from the genital tract. Urinary HE4 has been investigated in ovarian cancer diagnosis and a meta-analysis by Jia et al. suggested a promising pooled sensitivity of 76% and specificity of 92% [113]. However, there are no studies investigating the clinical utility of urinary HE4 for EC diagnosis or prognosis.

The anatomical continuity of the uterine cavity with the lower genital tract has also led to interest in cervico-vaginal samples as a source of biomarkers for EC. Most of the literature around urogenital sampling for EC has involved cytological diagnosis, obtaining cytology samples from either the cervix or the uterine cavity directly, with mixed accuracy [114]. A recent study by O’Flynn et al. reported a less invasive method of sampling using a Delphi screener, which does not require a full speculum examination, and reported pain scores similar to that of TVUS [112]. Urine and vaginal cytology had a sensitivity of 91.7% (95% CI 85–96.1) and specificity of 88.8% (95% CI 81.2–94.1) for EC detection in symptomatic women [115]. HE4 is present in the lower genital tract of healthy women. However, due to its role in immunity, the levels may be affected by the vaginal microbiome at the time of sampling [113]. There have been no studies investigating vaginal HE4 as a potential biomarker for EC, although work in this area is underway [114].

## 7. HE4 in Combination with Other Markers

The evidence for the clinical utility of serum HE4 in EC is promising. However, since ECs are a heterogenous group of tumours, it is possible that a combination of biomarkers may be of more value than HE4 alone. The most studied combination is HE4 and serum CA125.

CA125 is a well-established biomarker used for detection and recurrence monitoring of ovarian cancer but has also been shown to be raised in EC, although demonstrates less value as a single marker than HE4 [30,47]. The literature regarding the benefit of combining HE4 and CA125 for detection of EC is mixed. Several studies have shown a non-significant increase in sensitivity of the combination of serum HE4+CA125 for detection of EC compared to using HE4 alone [26,29,115], but this comes at a cost to specificity [38,48]. In contrast, other studies have shown no significant difference combining serum HE4 and CA125 compared to HE4 alone for EC detection [27,35]. Both these studies observed almost identical sensitivities for the combination of markers and for HE4 alone, with Angioli et al. demonstrating a sensitivities of 60% for both and Dewan et al. reporting sensitivities of 87% for both [27,35].

A limited number of studies have looked at the sensitivity of HE4 and CA125 in combination for detecting poor prognostic features such as LNM, with mixed results [67,68,73]. Wang et al. reported a higher sensitivity of HE4 and CA125 than HE4 alone for detection of LNM (94% vs. 82%), but the specificity was significantly lower (30% vs. 52%) [67]. In contrast, O’Toole et al. observed lower sensitivity but higher specificity of HE4 and CA125 for predicting LNM, LVSI, or MI > 50% compared to HE4 alone [68]. Only one study has reported on association of a combination of HE4 and CA125 with OS. Mutz-Dehbalaie et al. found that the combination of HE4 and CA125 was independently associated with OS and performed better than HE4 alone (HR 4.04, *p* = 0.023 vs. HR 2.407 *p* = 0.017) [62].

Overall, most studies suggest no benefit in the addition of CA125 to serum HE4, and it does not improve the accuracy of HE4 alone for the detection or prognosis of EC. It should be noted, however, that these studies are limited by their sample size, mixed study design and varying cut-offs used for serum HE4. In addition, the most frequent cut-off used for CA125 in the studies is 35 IU/L, which is that used for ovarian cancer detection, and may not be the optimal cut-off for EC.

Other biomarker combinations have been investigated for the diagnosis of EC with varying success. The addition of either soluble mesothelin-related peptide (SMRP) or the tumour marker CA72-4 to HE4 and CA125 proved to have no significant diagnostic benefit over HE4 alone [115]. A four marker panel consisting of HE4, CA125, CA72-4, and CA19-9 had a modest AUC of 0.82, but only performed marginally better than the three marker panel (HE4, CA125 and CA72-4, AUC 0.78) and HE4 alone (AUC 0.76) [36]. A panel of four markers including HE4, CA125, inflammatory apolipoprotein serum amyloid A (S-AA) and the protein carcinoembryonic antigen (CEA), demonstrated more promise, with a superior AUC (AUC 0.82 vs. 0.79) and sensitivity (84% vs. 75%) compared to HE4 alone [26]. Ge et al. investigated a four marker panel, including HE4, D-dimer, fibrinogen and CA199, for the risk stratification of women with abnormal vaginal bleeding, with an AUC of 0.88, and performed better than the combination of HE4 and CA125, and HE4 alone [40]. Possibly the best biomarker panel is that of HE4 and the transmembrane glycoprotein epithelial cell adhesion molecule (EpCAM). Lan et al. investigated several biomarkers for the diagnosis of EC including HE4, CA125, EpCAM and Transglutaminase 2 (TGM2) and found the combination of HE4 and EpCAM to provide the best AUC (0.87) and sensitivity (93%) [43]. Whilst these studies are promising, they may at best only produce marginal improvement in distinguishing patients with EC from benign or healthy endometrium compared to serum HE4 alone, and this would require validation in larger prospective cohorts.

Only one study has looked at a biomarker combination for predicting LNM. Gao et al. showed serum HE4 in combination with neutrophil-lymphocyte ratio (NLR) had a significantly higher sensitivity and specificity (97% and 96%, respectively) for predicting LNM compared to HE4 alone (87% and 74%, respectively) [83]. NLR could be a promising biomarker for increasing the accuracy of serum HE4 for predicting LNM, but more evidence is required to confirm this.

Algorithms and indices have been used to improve diagnostic risk stratification of patients with other cancers, such as the risk of malignancy index (RMI) and risk of ovarian malignancy algorithm (ROMA) used in ovarian cancer [116]. ROMA has been investigated as a risk stratifying tool for EC diagnosis and whilst it did not improve the overall diagnostic performance compared with HE4 (AUC 0.86 vs. 0.87 respectively), it did improve the sensitivity of HE4 both in the whole cohort and in pre-menopausal women [44]. Several studies have created algorithms or indices combining HE4, CA125 and other markers with clinical and/or imaging characteristics for diagnosis of EC. Knific et al. created an algorithm combining serum HE4, CA125 and BMI which discriminated between patients with EC and benign gynaecological conditions with a sensitivity of 67%, specificity of 85% and an AUC of 0.80, performing better than both HE4 alone (AUC = 0.77) or HE4 and CA125 combined (AUC = 0.79) [63]. Angioli et al. developed a risk stratification tool to triage women into high or low risk of EC by using data from patients with ultrasound-diagnosed endometrial abnormalities that were awaiting surgical intervention [117]. The Risk of Endometrial Malignancy (REM) index incorporates symptoms, serum HE4, endometrial thickness on ultrasound and age to create a percentage risk of EC. Using both a training and verification dataset, the REM score had an overall sensitivity and specificity of 92% and 96%, respectively, for distinguishing EC from benign endometrial diseases with an AUC of 0.96. This was externally validated using 298 patients by Plotti et al. and confirmed a sensitivity of 94% and a specificity of 95% [118]. This was developed further by adding BMI (REM-B) into the algorithm, which increased the sensitivity, specificity and AUC to 95%, 97% and 0.97, respectively [119]. The REM and REM-B were better than ultrasound pelvis alone for diagnosing EC which had a lower sensitivity and specificity of 81% and 61%, respectively [119]. These studies suggest that the incorporation of serum HE4 into algorithms that include factors, such as endometrial thickening, age, menopausal status and BMI, could improve the accuracy of serum HE4 for distinguishing EC from benign endometrial conditions, with REM and REM-B showing the most promising results. A recent study showed that the optimum cut-off for endometrial thickness on TVUS may vary according to race, which may need to be considered in algorithms that include TVUS [120].

Two studies have developed indices and/or algorithms incorporating serum HE4 and CA125 for use in prognosis of EC. Antonsen et al. created an index combining serum HE4, CA125 and age in patients with EC or AEH pre-operatively. This had a marginally higher AUC for predicting high risk features including MI >50%, >stage IA, LN positivity and CI, compared to serum HE4 alone [65]. Another study used a regression tree approach incorporating serum HE4, BMI and pre-operative stage. This had an accuracy for predicting patients with stage >I EC (at HE4 cut-off 81 pM) with an improved sensitivity, specificity and AUC (90%, 76%, 0.87 respectively) compared to that of serum HE4 alone (66%, 69%, 0.74, respectively) [121]. Although limited studies are available, indices combining serum HE4 and CA125 with demographic factors may improve accuracy of serum HE4 at predicting poorer prognostic factors and may provide useful in combination with molecular markers.

Table 5 and Table 6 summarise the performance of serum HE4 in combination with other biomarkers, or as part of indices, for the diagnosis and prognosis of EC, respectively.

## 8. Challenges of HE4 as a Biomarker

One of the main limitations for the utilisation of HE4 as a biomarker is that serum levels are significantly influenced by several physiological and demographic variables, making its interpretation more challenging. Several studies investigating the use of HE4 for EC have observed that serum HE4 levels increase with advancing age [25,60,61,74,124]. This has also been observed in studies investigating its use for ovarian cancer [125]. The association of HE4 levels with age has also been demonstrated in several population studies of healthy women in Europe and China [39,126,127]. Bolstad et al. reported that compared to women aged 20 years, the level of serum HE4 was 2%, 9%, 20%, 37%, 63% and 101% higher in women aged 40, 50, 60, 70 and 83 years, respectively [127]. Serum HE4 is also higher in postmenopausal women compared to those who are pre-menopausal [25,60,68,124]. However, this association may be secondary to the impact of age on HE4 levels rather than directly related to menopause. The risk of EC increases with advancing age, and the majority of women are therefore post-menopausal at diagnosis. Due to the impact of age on serum levels of HE4, it may not be appropriate for a single diagnostic or prognostic cut-off to be employed for all women. Furthermore, this would also present difficulties if HE4 were to be used for disease monitoring over a number of years, leading to increasing false positives. The use of age-adjusted cut-offs for HE4 specific to EC may be required to improve accuracy.

Serum levels of HE4 are significantly associated with renal impairment, which is the most common cause of raised HE4 levels in patients with benign gynaecological disease [127,128,129]. This may be due to the fact that HE4 is small in size and therefore cleared through glomerular filtration [130]. Due to the significant impact of eGFR on HE4 levels, several studies have suggested algorithms to adjust for this, both for the diagnosis of ovarian cancer and EC [131,132]. Chovanec et al. showed that in non-cancer patients, HE4 increases log-linearly with reduced eGFR <90 mL/min/1.73 m^2^ and developed a formula based on the patient’s renal function to produce an eGFR-adjusted HE4ren value [132]. They reported that whilst HE4 correlated with age, HE4ren did not (Spearman correlation coefficient 0.210 *p* = 0.0910), suggesting that the differences in HE4 levels seen with advancing age may actually be secondary to reduced eGFR, which is also associated with increasing age. They also demonstrated that the HE4ren value was superior to HE4 for the diagnosis of deep MI in EC in three different datasets. Caution is therefore required when interpreting the results of serum HE4 in the presence of renal impairment, and again, it is likely that modified cut-offs are recommended for those with significant renal disease. In particular, this may present a challenge in those undergoing adjuvant therapy where HE4 might be used for assessment of disease response, as renal function can worsen secondary to chemotherapy.

Several other factors influence serum HE4 levels, which may need to be considered. HE4 levels are 20–30% higher in smokers than non-smokers [39,126,127]. There have been limited studies investigating the relationship between HE4 and BMI. Reports are conflicting, with a number of studies reporting no correlation between serum HE4 and BMI [64,68,85,121], and others reporting a positive correlation [96] or an inverse correlation [131]. Bolstad et al. found that serum HE4 levels were lower in women with a BMI of 30 kg/m^2^ compared to those with a BMI of 25 kg/m^2^ [127]. Given the increasing prevalence of obesity and rising incidence of EC in younger patients, it would be important for age and BMI to be considered as potential confounders in future studies of HE4 as a biomarker. Unlike CA125, HE4 levels are not associated with endometriosis, although median serum HE4 levels are higher in women with pelvic inflammatory disease [133]. HE4 overexpression has also been reported in other non-gynaecological malignancies, including non-small cell lung cancer, pancreatic adenocarcinoma and transitional cell carcinoma [133].

Overall, the literature supports the potential clinical utility of HE4 for diagnosis, prognosis and surveillance for EC. However, there remain several important limitations. There is no consensus on which cut-off is best, and therefore there is significant heterogeneity seen in the studies presented, leading to challenges in interpretation and comparison of findings. Many studies use the cut-off of 70 pM or 140 pM, which are cut-offs developed and used for ovarian cancer diagnosis [134], and may be inappropriate for use in EC due to the differences in clinical, molecular and genomic behaviour of the two diseases. Other studies formulate their own optimum cut-offs based on AUC curves for patients in their study sample, which are relatively variable, or they use multiple cut-offs. As many of the studies are single centre with heterogenous populations, these customised cut-off values may not be optimal or applicable to different patient cohorts. In addition, there are likely to be different optimal cut-offs suitable for use in diagnosis, prognosis, prediction and recurrence. A number of different immunoassay techniques have been used to measure serum HE4 which do not always provide comparable results due to slight differences in the techniques and types of antibody used [135,136]. This not only makes it difficult when comparing findings, but presents a challenge should HE4 come into clinical use, as different laboratories use different analytical methods. The study designs are mixed, and include both prospective studies and retrospective studies, which could lead to selection bias, in particular for the prognostic ones. Most of the studies are single-centre and have small sample sizes. The use of molecular classification of EC is now increasingly being used in clinical practice. No studies to date have investigated serum HE4 in relation to EC molecular subgroups, and whether there might be an association that would aid diagnosis, prognosis and follow up planning, in particular for the MSI and NSMP groups, whose prognosis is very similar. Large multicentre prospective trials are now required to validate the clinical utility of HE4 and determine the most appropriate cut-offs. Its performance as a predictive biomarker for progestin treatment response in women undergoing non-surgical management of AEH and EC is of particular interest and warrants further study. Its use as a screening biomarker in asymptomatic high-risk women is another exciting area for development, especially when combined with patient-friendly, non-invasive biofluid sampling.

## 9. Conclusions

Serum HE4 is the most promising biomarker for EC to date, with potential roles in diagnosis, prognosis, prediction of hormone therapy response and recurrence monitoring. The combination of HE4 with CA125 or other biomarkers has so far shown only marginal improvements in utility. However, its use in indices or algorithms that incorporate imaging, physiological and/or demographic factors may have a greater impact on its performance. Its use as a clinical decision aid may allow a more personalised approach to the management of EC, so that women can avoid unnecessary investigations and treatments. Molecular classification of EC is likely to be employed more routinely in clinical practice over the coming years, and it will be necessary to investigate how HE4 might add to this for treatment and follow up strategies. Current challenges include a lack of agreement on the most appropriate serum cut-off values as well as the variation of serum HE4 according to physiological factors, including age and renal function. Larger multicentre clinical studies are required to confirm its utility as a biomarker, determine appropriate thresholds for use in EC and recommend adjustments to account for differences in physiological factors before it can be recommended for routine clinical use.

## Figures and Tables

**Table 1:** Serum HE4 cut-off values and performance for EC diagnosis.

Reference	C (*n*)	EC (*n*)	EEC (%)	Stage (%)	Comparison	HE4 Cut-Off (pM)	SE (%)	SP (%)	PV (%)	NV (%)	AUC
Abdalla et al., 2016 [32]	33	52	88%	I (71), II (6), III (23)	EC vs. benign/EH	70	73	86	88	68	0.88
						150	29	100	100	49	
						70 (preM)	29	97	94	48	
						150 (postM)					
Angioli et al., 2013 [35]	101	103	94%	I (49), II (12), III (36), IV(3)	EC vs. benign	70	60	100	100	72	0.86
					EC vs. benign	150	36	100	100	61	
Bian et al., 2017 [36]	87	105	81%	I (70), II (10), III (15), IV (5)	EC vs. healthy	140	58	-	60	67	0.76
Cymbaluk-Ploska et al., 2017 [37]	50	62	82%	I–II (81), III–IV (19)	EC (all) vs. healthy	70 (preM)	67	93	-	-	0.92
						140 (postM)					
					EC (preM) vs. benign	70	78	94	-	-	0.92
					EC (postM) vs. benign	140	64	84	-	-	0.86
Dewan et al., 2017 [27]	60	60	60%	I (70), II (20), III (10)	EC (postM) vs. healthy (postM)	70	87	100	-	-	0.97
Dong et al., 2017 [38]	200	150	-	I (49), II (23), III (20), IV (9)	EC vs. healthy/EH	92	57	96	90	77	0.82
Gasiorowska et al., 2016 [39]	46	46	78%	I (59), II (9), III (13), IV (2), U (15)	EC vs. benign	58	91	75	87	82	-
Ge et al., 2020 [40]	31	127	-	-	EC vs. AEH/healthy	59	95	31	-	-	0.79
Jafari-Shobeiri et al., 2016 [41]	60	40	88%	I (53), II (33), III (15)	EC vs. benign	70	58	93	85	77	0.82
Kemik et al., 2016 [42]	50	50	72%	I (66), II (22), III (12)	EC vs. healthy	36	94	36	-	-	0.88
						39	90	42	-	-	
Lan et al., 2020 [43]	84	42	-	-	EC vs. benign/healthy	49	71	84	70	86	0.83
					EC vs. healthy	45	81	91	89	83	0.93
					EC vs. benign	54	62	78	74	67	0.72
Li et al., 2016 [44]	727	147	-	I–II (76), II–IV (22), U (1)	EC vs. benign	70 (preM)	44	98	82	90	0.93
						140 (postM)					
					EC (preM) vs. benign	70	57	98	75	95	0.92
					EC (postM) vs. benign	140	32	100	100	64	0.92
Liu et al., 2015 [30]	100	93	71%	I (58), II (19), III (23)	EC vs. healthy	142	62	95	-	-	-
					EEC vs. healthy	142	48	95	-	-	0.8
					Serous papillary vs. healthy	142	89	95	-	-	0.99
Liu et al., 2021 [45]	136	127	96%	I (82), II (4), III (13), IV (1)	EC vs. benign	52	57	76	72	63	0.70
Minar et al., 2016 [46]	150	150	100%	I (78), II (7), III (11), IV (5)	EC (stage I–IV) vs. benign	49	88	57	69	81	0.77
						99	37	93	86	58	
					EC (stage I–III) vs. benign	49	86	57	64	81	0.74
						99	29	93	80	59	
Omer et al., 2013 [26]	35	64	84%	I (94), II–III (6)	EC vs. healthy	60	75	66	83	54	0.78
Presl et al., 2014 [47]	32	34	-	I (76), II (18), III (6)	EC vs. healthy	90	41	97	93	61	0.81
						62	56	91	86	66	
						73	53	97	95	66	
						49	79	78	79	78	
Yilmaz et al., 2017 [48]	40	26	96%	I (85), II (4), III (12)	EC vs. healthy	63	58	78	-	-	0.63
Yilmaz et al., 2016 [31]	90	77	85%	I (80), II (10), III–IV (10)	EC vs. benign	61	73	84	80	78	0.87
Zanotti et al., 2012 [49]	125	193	79%	I (55), II (18), III (16), IV (5), U (7)	EC (stage I–IV) vs. healthy	56	74	90	-	-	0.88
						64	66	95	-	-	
						85	46	99	-	-	
					EC (stage I) vs. healthy	57	57	90	-	-	0.82
						64	53	95	-	-	
						85	32	99	-	-	
					EC (stage II–IV) vs. healthy	56	91	90	-	-	0.95
						63	82	95	-	-	
						85	65	99	-	-	

*n* (number of patients), C (controls), EC (endometrial carcinoma), EEC (endometrioid endometrial carcinoma), HE4 (human epididymis protein 4), U (unknown), SE (sensitivity), SP (specificity), PV (positive predictive value), NV (negative predictive value), AUC (receiver-operating characteristic area under curve), EH (endometrial hyperplasia), AEH (atypical endometrial hyperplasia), preM (premenopausal), postM (postmenopausal).

**Table 2:** Serum HE4 cut-off values and performance for EC prognostic factors.

Reference	EC	EEC	Stage (%)	Comparison	HE4 Cut-Off (pM)	SE (%)	SP (%)	PV (%)	NV (%)	AUC
Abdalla et al., 2016 [82]	52	89%	I (70), II (8), III (23)	Stage IB–IIIC vs. IA	70	85	47	-	-	-
				Stage II–III vs. I	70	81	31	-	-	-
				Stage IIIC vs. IA–IIIB	70	100	29	-	-	-
Abbink et al., 2018 [64]	157	78%	I (63), II (7), III (17), IV (14)	LNM vs. no LNM	130	65	79	-	-	0.72
Antonsen et al., 2013 [65]	352	84%	AEH (5), I (67), II (11), III (14), IV (2)	Stage IB vs. AEH/IA	70	55	69	-	-	0.68
				EC vs. AEH	70	44	77	-	-	0.64
				MI > 50% vs. ≤50%	70	60	68	-	-	0.70
				LNM vs. no LNM	70	76	49	-	-	0.70
				High vs. Low/Medium risk	70	50	66	-	-	0.57
				CI vs. no CI	70	63	63	-	-	0.69
Brennan et al., 2014 [74]	373	85%	I (85), II (8), III (6), IV (1)	MI > 50% vs. ≤50%	70	83	53	34	92	0.76
				MI > 50% vs. ≤50% (EEC)	70	84	54	33	92	0.76
				MI > 50% vs. ≤50% (G1/2 EEC)	70	84	55	32	93	0.77
				MI > 50% vs. ≤50% (Stage 1–2 EEC)	70	83	55	29	94	-
Capriglione et al., 2015 [71]	232	94%	I(69), II(9), III(17), IV(5)	Stage IA vs. other	61	82	96	92	91	-
				Stage IB vs. other	89	83	96	92	92	-
				Stage II vs. other	104	81	99	89	97	-
				Stage III vs. other	153	93	99	95	98	-
				Stage IV vs. other	204	82	99	90	99	-
Dobrzycke et al., 2016 [73]	78	100%	I (91), II (9)	Lymphadenectomy vs. no lymphadenectomy (postM)	78	87	67	89	51	0.81
Gasiorowska et al., 2016 [39]	46	78%	I (59), II (9), III (13), IV (2), U (15)	Lymphadenectomy vs. no lymphadenectomy	77	76	75	-	-	-
Gao et al., 2021 [83]	-	145	I–II (83), III–IV (17)	LNM vs. no LNM	80	87	74	-	-	0.71
Knific et al., 2017 [63]	64	89%	I (84), III (11), IV (3)	MI > 50% vs. ≤50%	176	87	67	45	67	0.78
				LVSI vs. no LVSI	176	67	82	38	82	0.81
Liu et al., 2021 [45]	136	96%	I (82), II (4), III(13), IV (1)	Stage I vs. II–IV	37	28	87	-	-	0.49
Minar et al., 2015 [59]	115	100%	I (78), II (7), III (12), IV (5)	Stage IA vs. IB	78	70	68	89	39	0.70
				Stage IA vs. IB–II	78	68	68	84	46	0.70
				Stage IA vs. IB–III	110	59	87	78	73	0.76
				Stage IA vs. IB–IV	110	61	87	77	76	0.77
				Stage IA vs. IB	239	10	94	78	33	0.70
				Stage IA vs. IB–II	239	11	94	72	43	0.70
				Stage IA vs. IB–III	239	22	94	67	69	0.76
				Stage IA vs. IB–IV	239	24	94	65	73	0.77
				Low vs. high risk	77	72	75	75	73	0.77
				Low vs. high risk	242	19	95	54	79	0.77
O’Toole et al., 2021 [68]	147	100%	I (75), II (11), III (12), IV (2)	LNM vs. no LNM	81	79	53	15	96	0.66
				MI > 50% vs. ≤50%	81	67	60	47	77	0.63
				LVSI	81	61	54	32	80	0.57
Presl et al., 2017 [84]	124	89%	I (94), III (8)	High/intermediate risk vs. low risk	113	40	84	-	-	-
				High vs. low/intermediate risk	115	36	75	-	-	-
Prueksaritanond et al., 2016 [70]	70	-	I (70), II (13), III (14), IV (3)	High vs. low risk	70	83	80	96	44	0.88
Panyavaranant et al., 2020 [55]	128	98%	I (67), II (12), III (15), IV (6)	High vs. low risk	113	64	77	74	68	0.70
Rajadevan et al., 2021 [85]	100	84%	I (79), II (8), III (12), IV (1)	High grade/MI > 50% vs. low grade/MI ≤ 50%	70	75	50	32	86	-
Romera et al., 2020 [69]	126	100%	I–II (91), III–IV (10)	Stage III–VI vs. I–II	156	67	90	40	96	0.84
				LNM vs. no LNM	156	71	88	25	98	0.80
Stiekema et al., 2017 [66]	88	64%	I (68), II–IV (32)	MI > 50% vs. ≤50%	60 (<40 years)	67	64	40	84	-
					75 (40–60 years)					
					90 (>60 years)					
					70	89	56	42	93	
Wang et al., 2017 [67]	258	-	I (86), II (5), III–IV (10)	LNM vs. no LNM	73	83	52	11	98	-
Zamani et al., 2019 [86]	131	88%	I (77), II (12), III (11)	Stage IA vs. IB–IIIC	70	64	60	-	-	-
					140	34	100	-	-	-
				Stage I vs. II–IIIC	70	63	53	-	-	-
					140	43	100	-	-	-

EC (endometrial carcinoma), EEC (endometrioid endometrial carcinoma), HE4 (human epididymis protein 4), SE (sensitivity), SP (specificity), PV (positive predictive value), NV (negative predictive value), AUC (receiver-operating characteristic area under curve), LNM (lymph node metastases), AEH (atypical endometrial hyperplasia), CI (cervical invasion), MI (myometrial invasion), LVSI (lymphovascular space invasion), G (grade).

**Table 3:** Serum HE4 cut-off value and performance for LNG-IUS response.

Reference	*n*	Stage	Comparison	HE4 Cut-Off (pM)	SE (%)	SP (%)	AUC
Behrouzi et al., 2020 [96]	49	AEH/IA EEC	Non-responder vs. responder to LNG-IUS	165	39	100	0.76

*n* (number of patients), HE4 (human epididymis protein 4), EEC (endometrioid endometrial carcinoma), LNG-IUS (levonorgestrel-releasing intrauterine system), AEH (atypical endometrial hyperplasia), SE (sensitivity), SP (specificity), AUC (receiver-operating characteristic area under curve).

**Table 4:** Serum HE4 cut-off values and performance for EC recurrence.

Reference	EC	EEC	Stage (%)	Comparison	HE4 Cut-Off (pM)	SE (%)	SP (%)	PV (%)	NV (%)	AUC
Brennan et al., 2015 [104]	98	70%	I (59), II (7), III (27), IV (8)	Recurrence vs. no recurrence (EC)	70	81	64	45	90	0.81
				Recurrence vs. no recurrence (EEC)	70	84	74	55	93	0.87
Angioli et al., 2016 [109]	252	95%	I (65), II (10), III (18), IV (7)	Recurrence vs. no recurrence (5 year follow up) (EC)	70	67	53	71	91	-
					201	80	91	90	81	-
				Recurrence vs. no recurrence (5 year follow up) (EEC)	70	74	61	78	68	-
					201	83	95	91	95	-

HE4 (human epididymis protein 4), EC (endometrial carcinoma), EEC (endometrioid endometrial carcinoma), SE (sensitivity), SP (specificity), PV (positive predictive value), NV (negative predictive value), AUC (receiver-operating characteristic area under curve).

**Table 5:** HE4 biomarker combinations, cut-off values and their performance in diagnosis of EC.

Reference	C (*n*)	EC (*n*)	EEC (%)	Stage	HE4 Cut-Off (pM)	Other Cut-Off	Comparison	Combination/Index	SE (%)	SP (%)	PV (%)	NV (%)	AUC
Angioli et al., 2013 [35]	103	101	94%	I (49), II (12), III (36), IV (3)	70	CA125 = 35 U/mL	EC vs. benign	HE4, CA125	60	100	-	-	-
					150				35	100	-	-	-
Angioli et al., 2013 [117]	391	60	97%	I (77), II(12), III(8), IV (3)	-	REM 0.3185	EC vs. benign	REM (symptom, HE4, US endometrial thickness, age)	94	97	83	98	0.96
	196	28	96%	I (82), II (7), III (7), IV (4)	-	REM 0.3185			89	95	73	98	0.92
Benati et al., 2016 [122]	29	45	91%	I (69), II (16), III (13), IV (1)	64	DJ-1 = 3654 pg/mL	EC vs. healthy	HE4, DJ-1	-	-	-	-	0.96
Bian et al., 2017 [36]	87	105	89%	I (70), II (10), III (15), IV (5)	140	CA125 = 35 U/mL CA724 = 6.9 U/mL, CA19-9 = 37 U/mL	EC vs. healthy	HE4, CA125, CA724	58	-	70	79	0.78
								HE4, CA125, CA19-9	56	-	74	82	0.79
								CA19-9, CA125, CA724	41	-	65	80	0.67
								HE4, CA19-9, CA724	55	-	54	76	0.79
								HE4, CA125, CA724, CA19-9	59	-	88	90	0.82
							EC (I) vs. healthy	HE4, CA125, CA724	-	-	-	-	0.72
								HE4, CA125, CA19-9	-	-	-	-	0.75
								CA19-9, CA125, CA724	-	-	-	-	0.61
								HE4, CA19-9, CA724	-	-	-	-	0.71
								HE4, CA125, CA724, CA19-9	-	-	-	-	0.77
							EC (II-IV) vs. healthy	HE4, CA125, CA724	-	-	-	-	0.84
								HE4, CA125, CA19-9	-	-	-	-	0.83
								CA19-9, CA125, CA724	-	-	-	-	0.73
								HE4, CA19-9, CA724	-	-	-	-	0.82
								HE4, CA125, CA724, CA19-9	-	-	-	-	0.90
Dewan et al., 2017 [27]	60	62	60%	I (70), II (20), III (10)	70	CA125 = 35 U/mL	EC vs. healthy (postM)	HE4, CA125	87	100	-	-	-
Plotti et al., 2017 [118]	196	102	81%	-	-	-	EC vs. benign	REM	94	95	91	95	-
Plotti et al., 2018 [119]	391	60	97%	I (77), II (12), III (8), IV (3)	-	REM-B 0.3925	EC vs. benign	REM-B (REM, BMI)	97	98	90	99	0.97
	196	28	96%	I (82), II (7), III (7), IV (4)	-	REM-B 0.3925	EC vs. benign		93	96	78	99	0.93
Vezzoli et al., 2017 [121]	-	293	87%	I (66), II–IV (34),	-	-	EC ≤ I vs. >I	RERT (HE4, CA125, age, BMI, children number, menopausal status, contraception, HRT, hypertension, grading, clinical stage)	90	76	65	94	0.87
Dong et al., 2017 [38]	200	150	-	I (49), II (23), III(20), IV (9)	92	CA125 = 31 KU/L	EC vs. benign/healthy	HE4, CA125	73	91	85	83	0.79
Knific et al., 2017 [63]	69	64	89%	I (84), III (11), IV (3)	176	-	EC vs. benign	diagnostic model (HE4, CA125, BMI)	67	85	81	85	0.80
						CA125 = 34 kU/L		HE4, CA125	-	-	-	-	0.79
Ge et al., 2020 [40]	31	223	-	AEH (43), EC (57)	59	-	EC vs. AEH/healthy	HE4, CA125	95	33	-	-	0.80
						Risk index of EC (RIEC) = 0.36		RIEC (D-dimer, fibrinogen, HE4, CA19-9)	95	66	-	-	0.88
Lan et al., 2020 [43]	41	84	-	-	49	EpCAM = 205 pg/mL CA125 = 17 U/mL	EC vs. benign/healthy	HE4, CA-125, EpCAM	83	77	65	90	0.88
								HE4, EpCAM	93	69	60	95	0.87
								HE4, CA125	74	80	65	86	0.83
					45	EpCAM = 205 pg/mL CA125 = 17 U/mL	EC vs. healthy	HE4, CA-125, EpCAM	93	93	93	93	0.96
								HE4, EpCAM	93	93	93	93	0.96
								HE4, CA125	81	93	81	93	0.93
					54	EpCAM = 204 pg/mL CA125 = 17 U/mL	EC vs. benign	HE4, CA-125, EpCAM	71	78	77	72	0.80
								HE4, EpCAM	64	81	77	69	0.79
								HE4, CA125	41	100	41	100	0.72
Liu et al., 2018 [29]	30	40	-	I (33), II (55), III (10), IV (3)	52	CA125 = 35 U/mL	EC vs. benign/healthy	HE4, CA125	63	-	-	-	-
Omer et al., 2013 [26]	34	64	83%	I (94), II-III (6)	60	S-AA = 8.8 U/mL CEA = 1.4 ng/mL CA125 = 14.2 U/mL	EC vs. healthy	HE4, CA125	78	72	88	57	0.78
								HE4, CEA	82	73	89	59	0.79
								HE4, S-AA	73	64	83	50	0.75
								HE4, S-AA, CEA, CA125	84	61	86	58	0.82
Zanotti et al., 2012 [49]	125	193	79%	I (55), II (18), III (16), IV (5), U (13)	56	CA125 = 12 U/mL	EC vs. healthy	HE4, CA125	79	90	-	-	-
					51	CA125 = 34 U/mL			64	95	-	-	-
					77	CA125 = 16 U/mL			57	99	-	-	-
					56	CA125 = 12 U/mL	EC (stage I) vs. healthy		68	90	-	-	-
					69	CA125 = 12 U/mL			52	95	-	-	-
					53	CA125 = 43 U/mL			42	99	-	-	-
					54	CA125 = 15 U/mL	EC (stage II–IV) vs. healthy		93	90	-	-	-
					60	CA125 = 26 U/mL			77	95	-	-	-
					89	CA125 = 7 U/mL			69	99	-	-	-

*n* (number of patients), C (controls), EC (endometrial carcinoma), EEC (endometrioid endometrial carcinoma), HE4 (human epididymis protein 4), CA125 (cancer antigen 125), BMI (body mass index), EpCAM (epithelial cell adhesion molecule), S-AA (serum amyloid A), CA724 (cancer antigen 724), CA19-9 (cancer antigen 19-9), CEA (carcinoembryonic antigen), risk of endometrial malignancy (REM), representative regression tree (RERT), RIEC (risk index of endometrial cancer), AEH (atypical endometrial hyperplasia), HRT (hormone replacement therapy), SE (sensitivity), SP (specificity), PV (positive predictive value), NV (negative predictive value), AUC (receiver-operating characteristic area under curve).

**Table 6:** HE4 biomarker combinations, cut-off values and their performance in prognosis of EC.

Reference	EC (*n*)	EEC (%)	Stage (%)	HE4 Cut-Off (pM)	Other Cut-Off	Comparison	Combination	SE (%)	SP (%)	PV (%)	NV (%)	AUC
Angioli et al., 2016 [123]	38	2%	I (69), II (19), III (11), IV (3)	63	-	MI ≥ 50% vs. <50%	HE4, TVUS	88	96	93	92	-
							HE4, dc MRI	86	96	92	92	-
							HE4, TVUS, dc MRI	100	96	93	100	-
	41	5%	I (78), II (15), III (5), IV (2)		-		HE4, TVUS	96	100	100	93	-
							HE4, dw MRI	97	100	100	88	-
							HE4, TVUS, dw MRI	96	100	100	93	-
	38	2%	I (69), II (19), III (11), IV (3)	108	-	CI vs. no CI	HE4, TVUS	100	100	-	-	-
							HE4, dc MRI	100	96	93	100	-
							HE4, TVUS, dc MRI	100	96	92	100	-
	41	5%	I (78), II (15), III (5), IV (2)		-		HE4, TVUS	100	100	-	-	-
					-		HE4, dw MRI	92	96	92	96	-
					-		HE4, TVUS, dw MRI	100	100	100	100	-
Antonsen et al., 2013 [65]	352	84%	AEH (5), I (68), II (11), III (14), IV (2)	-	-	MI ≥ 50% vs. <50%	Index (HE4, age, CA125)	75	38	-	-	0.74
								85	25	-	-	
								95	19	-	-	
						LNM vs. no LNM		75	55	-	-	0.78
								85	51	-	-	
								95	23	-	-	
						CI vs. no CI		75	48	-	-	0.72
								85	38	-	-	
								95	10	-	-	
						High vs. low/medium risk		-	-	-	-	0.66
						EC vs. AEH		-	-	-	-	0.66
						Stage IB vs. AEH/IA		-	-	-	-	0.70
Dobrzycke et al., 2016 [73]	78	100%	I (91), II (9)	78	CA125 = 26 U/mL	Lymphadenectomy vs. no lymphadenectomy (postM)	HE4, CA125	81	66	48	83	-
Gao et al., 2020 [83]	145	81%	I–II (83), III–IV (17)	80	NLR = 2.5	LNM vs. no LNM metastasis	HE4, NLR	97	96	-	-	-
O’toole et al., 2021 [68]	147	100%	I (75), II (11), III (12), IV (2)	81	CA125 = 35 U/mL	LNM vs. no LNM	HE4, CA125	43	94	43	94	0.68
						MI ≥ 50% vs. <50%		20	96	71	70	0.58
						LVSI		25	95	64	79	0.60
Wang et al., 2017 [67]	258	-	I (86), II (5), III–IV (10)	73	CA125 = 13.5 U/mL	LNM vs. no LNM	HE4, CA125	94	31	9	99	-

*n* (number of patients), HE4 (human epididymis protein 4), CA125 (cancer antigen 125), LNM (lymph node metastasis), MI (myometrial invasion), CI (cervical invasion), EpCAM (epithelial cell adhesion molecule), LVSI (lympho-vascular space invasion), C (controls), EC (endometrial carcinoma), EEC (endometrioid endometrial carcinoma), SE (sensitivity), SP (specificity), PV (positive predictive value), NV (negative predictive value), AUC (receiver-operating characteristic area under curve), US (ultrasound), postM (postmenopausal), BMI (body mass index), TVUS (transvaginal ultrasound), dw MRI (diffusion weighted MRI), dc MRI (double-contrast MRI), AEH (atypical endometrial hyperplasia), NLR (neutrophil-lymphocyte ratio)
